# A taxonomic review of the Hydraenidae in South Korea (Coleoptera)

**DOI:** 10.3897/zookeys.634.10640

**Published:** 2016-11-21

**Authors:** Dae-Hyun Lee, Kee-Jeong Ahn

**Affiliations:** 1 Institute of Ecosystem Restoration Planning, 97 Deokmyeong-ro, Yuseong-gu, Daejeon 34155, Republic of Korea; 2 Department of Biology, Chungnam National University, Daejeon 34134, Republic of Korea

**Keywords:** Coleoptera, Hydraenidae, South Korea, taxonomy

## Abstract

A taxonomic study of the South Korean Hydraenidae is presented. Eight species in two genera are recognized, one of which is reported for the first time in the Korean peninsula, *Ochthebius marinus* (Paykull). It was also found that *Hydraena riparia* Kugelann and *Ochthebius inermis* Sharp previously recorded in South Korea were incorrect identifications of *Hydraena puetzi* Jäch and *Ochthebius lobatus* Pu, respectively. Habitus and SEM photographs, line drawings of aedeagus, distribution maps, keys, and redescriptions of the species are provided.

## Introduction

The Hydraenidae, relatively small water beetles with unique habitus, are usually found at margins of running water, slowly flowing water, and stagnant water in the vicinity of lotic biotopes (Jäch et al. 2016). Approximately 1600 species in 42 genera have been recorded in the world (Ślipiński et al. 2011); 941 species in nine genera in the Palaearctic (Jäch and Skale 2015); 100 species in seven genera in China, 35 species in three genera in Japan; and 11 species in three genera in the Far East of Russia (Jäch and Skale 2015).

Members of the Hydraenidae are characterized by combination of the following features: separation of the gula and submentum by confluent genae; presence of an anterior plate-like premento-hypopharyngeal extension; large labrum with a deep medial incision in some species; and palpigers connected by a transverse sclerotized bar (Beutel et al. 2003).

Kwon and Suh (1986) first recorded *Hydraena riparia* Kugelann in South Korea. Later, Lee (1995) added two species (*Ochthebius inermis* Sharp and *Ochthebius satoi* Nakane) with descriptions and habitus illustrations. Recently, Jäch and Delgado (2014) found that a marine littoral species of *Neochthebius granulosus* (Satô) previously recorded by Park and Ahn (2008) was an incorrect identification of *Ochthebius ahni* Jäch and Delgado and *Ochthebius parki* Jäch and Delgado. Jäch and Skale (2015) added four species [*Hydraena miyatakei* Satô, *Hydraena puetzi* Jäch, *Ochthebius hasegawai* Nakane and Matsui, and *Ochthebius lobatus* Pu]. Accordingly, nine species in two genera have been recorded in South Korea.

In this paper, one species is reported for the first time in the Korean peninsula, *Ochthebius marinus* (Paykull). It was also found that *Hydraena riparia* and *Ochthebius inermis* previously recorded in South Korea were incorrect identifications of *Hydraena puetzi* and *Ochthebius lobatus*, respectively. Habitus and scanning electron microscopy photographs, keys, redescriptions, and diagnostic characters with illustrations of the species are provided.

## Material and methods

To identify South Korean hydraenid species more reliably, they were compared with voucher specimens in the Natural History Museum (**NHM**, London, United Kingdom), Naturhistorisches Museum (**NMW**, Wien, Austria) and Ehime University Museum (**EUMJ**, Matsuyama, Japan). The specimens used in this study are deposited in Chungnam National University Insect Collection (**CNUIC**, Daejeon, Korea). Habitus and scanning electron microscope (SEM) photographs were prepared based on a former study (Lee and Ahn 2015). See Jäch and Delgado (2014) for detailed descriptions of *Ochthebius ahni* and *Ochthebius parki*. The terms of taxonomic characters and measurements of specimens mainly followed Perkins (2001) and Jäch et al. (2016). The geographical subdivision of China and Russia was based on the standards of Löbl and Löbl (2015).

## Results

### Hydraenidae Mulsant, 1844

#### Key to the genera of South Korean Hydraenidae

**Table:** 

1	Anterior margin of mentum (Fig. 8) dentate; maxillary palpi longer than antenna	***Hydraena***
–	Anterior margin of mentum (Fig. 18) not dentate; maxillary palpi shorter than antenna	***Ochthebius***

#### Genus Hydraena

Taxon classificationAnimaliaColeopteraHydraenidae

Kugelann, 1794

Hydraena Kugelann, 1794: 578. Type species Hydraena riparia Kugelann, 1794.

##### Diagnosis.

Labral-mandibular interlocking device present. Mentum (Fig. 8) with acute median projection on anterior part. Sensilla various and complex. Features of the secretion delivery system specialized (Jäch et al. 2000).

##### Key to the subgenera of South Korean *Hydraena*

**Table:** 

1	Body brown to dark blue; longitudinal median genal suture and longitudinal inner genal carina complete	***Hydraena***
–	Body yellowish brown; longitudinal median genal suture and longitudinal inner genal carina incomplete or absent	***Hydraenopsis***

#### Subgenus Hydraena

Taxon classificationAnimaliaColeopteraHydraenidae

Kugelann, 1794

Hydraena Kugelann, 1794: 578. Type species: Hydraena riparia Kugelann, 1794. See Jäch and Skale (2015) for more detailed synonymy and references.

##### Diagnosis.

Lateral margin of labrum (Fig. 6) abruptly constricted posteriorly. Setae of labial palpomere 2 closely set. Anterior apex of modified longitudinal hypomeral carina with antennal pocket setae. Elytra (Fig. 11) with 15 or more striae. Mesoventral process (Fig. 14) obtuse, between 100° and 130° angle. Sternite III (Fig. 17) without pubescence behind coxal pits. Sternite VII (Fig. 17) pubescent with semicircular glabrous posterior region. Gonocoxite with a pair of subapical tufts (Jäch et al. 2000).

#### Hydraena (Hydraena) puetzi

Taxon classificationAnimaliaColeopteraHydraenidae

Jäch, 1994

Figs 1 , 6–17 , 50 , 55

Hydraena (*s. str.*) puetzi Jäch, 1994: 43; Jäch and Skale 2015: 139.

##### Specimens examined.

**SOUTH KOREA**: Gangwon Prov.: 1♀, Goseong-gun, Ganseong-eub, Jinburyeong, N38°15'59" E128°23'04", 640m, 21.VII.2004, KM Yang, JS Park, leaf litter; 1♂ 2♀♀,Hongcheon-gun, Nae-myeon, Changchon-ri, Unduryeong-hill, 10.VIII.2012, SW Jeong, mountain stream; 1♂, Hongcheon-gun, Seo-myeon, Bangok-ri, Bangokgyo, 17.IX.2009, DH Lee, mountain stream (1♂, on slide); 1♂ 3♀♀, Pyeongchang-gun, Daegwanryeong-eub, Yucheon-ri, Haewon-temple, 15.IX.2009, YJ Park, under stone on stream margin (1♂, on slide); 1♀, Pyeongchang-gun, Jinbu-myeon, Dongsan-ri, Mt. Odaesan, Sangwon-temple, 16.VIII–15.IX. 2001, SJ Park, CW Shin, FIT; 1♂, same data as former except for, 8.V.2004, DH Lee, stream; 1♂, same data as former except for, 8.V.2004, DH Lee, stream; 9♂♂ 11♀♀, same data as former except for, Mt. Odaesan, 22.V.2012, DH Lee, springfed pool; 1♂, Samcheok-si, Gagok-stream, 25.VI.1985. SH Lee; 3♀♀, Yeongwol-gun, Suju-myeon, Mt. Baehyang-san, 17.VII.2010, SW Jeong, mountain stream; Gyeongbuk Prov.: 1♀, Uljin-gun, Buk-myeon, Mt. Eungbongsan, 6.VI.1995, SH Lee; 1♂, Uljin-gun, Seo-myeon, Wangpi-ri, Golan-bridge, N36°54'27.1" E129°14'34.2", 385m, 26.IV.2012, DH Lee, mountain stream; Gyeonggi Prov.: 1♀, Gapyeong-gun, Buk-myeon, Jeongmok-ri, Garim-bridge, 16.IX.2010, SW Jeong, stream; 1♂, Paju-si, Jangdan-myeon, Nosang-ri, 21.IX.2012, SW Jeong, stream.

##### Published South Korean records.

*Hydraena puetzi*: Jäch and Skale (2015: 103). *Hydraena riparia*: Kwon and Suh (1986: 99); Kim et al. (1994: 134); Lee (1995: 13); Han et al. (2007: 271); Han et al. (2008: 263); Cho and Park (2010: 96) [misidentification].

##### Redescription.

Length 2.0–2.3 mm. Head black; pronotum and elytra brown to dark blue; ventral surface dark brown. Head (Fig. 6) trapezoidal, widest cross eyes, ventral side (Fig. 7) with sparse setae. Anterior margin of labrum (Fig. 6) nearly straight except antero-medial part deeply excised. Clypeus (Fig. 6) with relatively small punctures; antero-medial margin broadly rounded; antero-lateral part protruded. Frontoclypeal suture (Fig. 6) bisinuate. Mentum (Fig. 8) subquadrate, widest at posterior corner, with large punctures on anterior and lateral parts; anterior margin broadly rounded, with a row of thick setae; posterior part protruded laterally. Submentum (Fig. 7) semicircular; antero-medial margin nearly straight, dentate; antero-lateral margin protruded and acute. Antenna with nine antennomeres; 1 longest, approx. 2.5 times as long as 2; 2 approx. 6.0 times as long as 3, bulbous at basal part; 3 bulbous at apical part; 4 dish-shaped; 5–9 clubbed and with pubescence. Maxillary palpomere (Fig. 9) 1 smallest; 2 longest, approx. 3.0 times as long as 3, few setae present on ventral part, with transverse imbricate reticulation except apical part; 3 bulbous at apical part, few setae present on dorsal and ventral parts, with imbricate reticulation except anterior third; 4 approx. 2.0 times as long as 3, many setae present on dorsal and ventral parts, apical part acute, with imbricate reticulation except anterior to middle. Pronotum (Fig. 10) hexagonal, widest at middle and narrowest posterior part, medial part protruded and flattened; anterior margin sinuate; antero-median margin straight; anterior and posterior corners rectangular; lateral margin rounded and serrated. Elytra (Figs 11, 12) widest at middle; antero-lateral and postero-lateral parts serrated. Prosternum (Fig. 13) transverse, with longitudinal carina on medial part; anterior and posterior corners acute. Mesoventrite (Fig. 14) reverse pentagonal, with two vertical carina on each side of midline. Mesotibia (Fig. 15) serrate on apico-lateral part, with thick spines. Metaventrite (Fig. 16) flattened, with two glabrous parts medially. Sternite VII (Fig. 17) with many long setae on medial part in female. Male terminal sternite semicircular; postero-medial part bifid; posterior part rounded and with many setae. Median lobe of aedeagus (Fig. 50) longer than paramere, strongly curved at middle; a seta present on anterior third; apical part acute and protruded; flagellum bisinuate. Left paramere (Fig. 50) as long as right; apical part long-oval; many long setae present on apical margin. Apical part of right paramere (Fig. 50) semicircular; many long setae present on apical margin.

**Figures 1–5.**
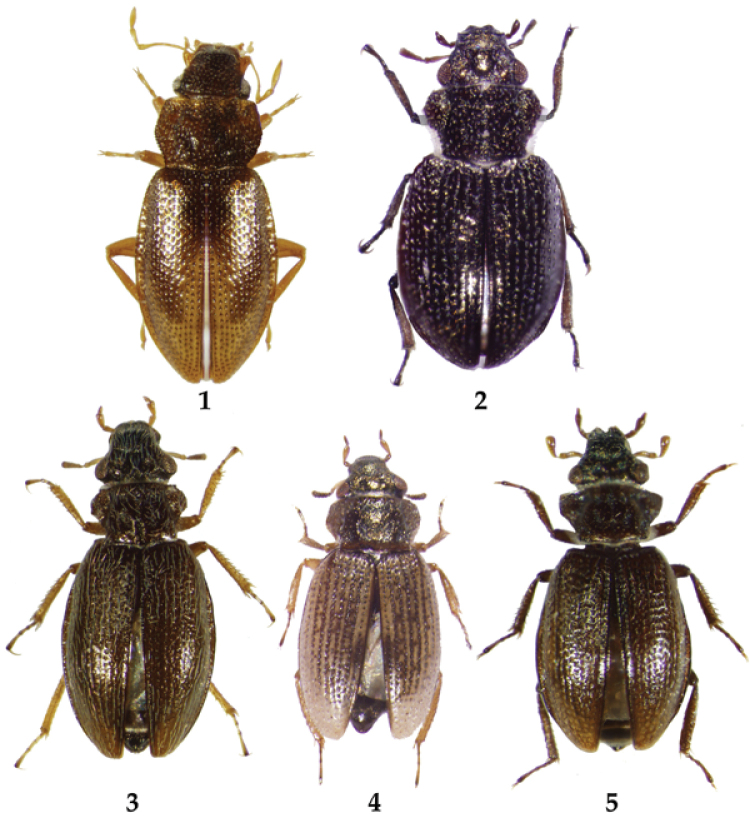
Habitus. **1** *Hydraena puetzi*, 2.2 mm **2** *Ochthebius hasegawai*, 1.8 mm **3** *Ochthebius lobatus*, 2.2 mm **4** *Ochthebius marinus*, 2.0 mm **5** *Ochthebius satoi*, 1.8 mm.

**Figures 6–17.**
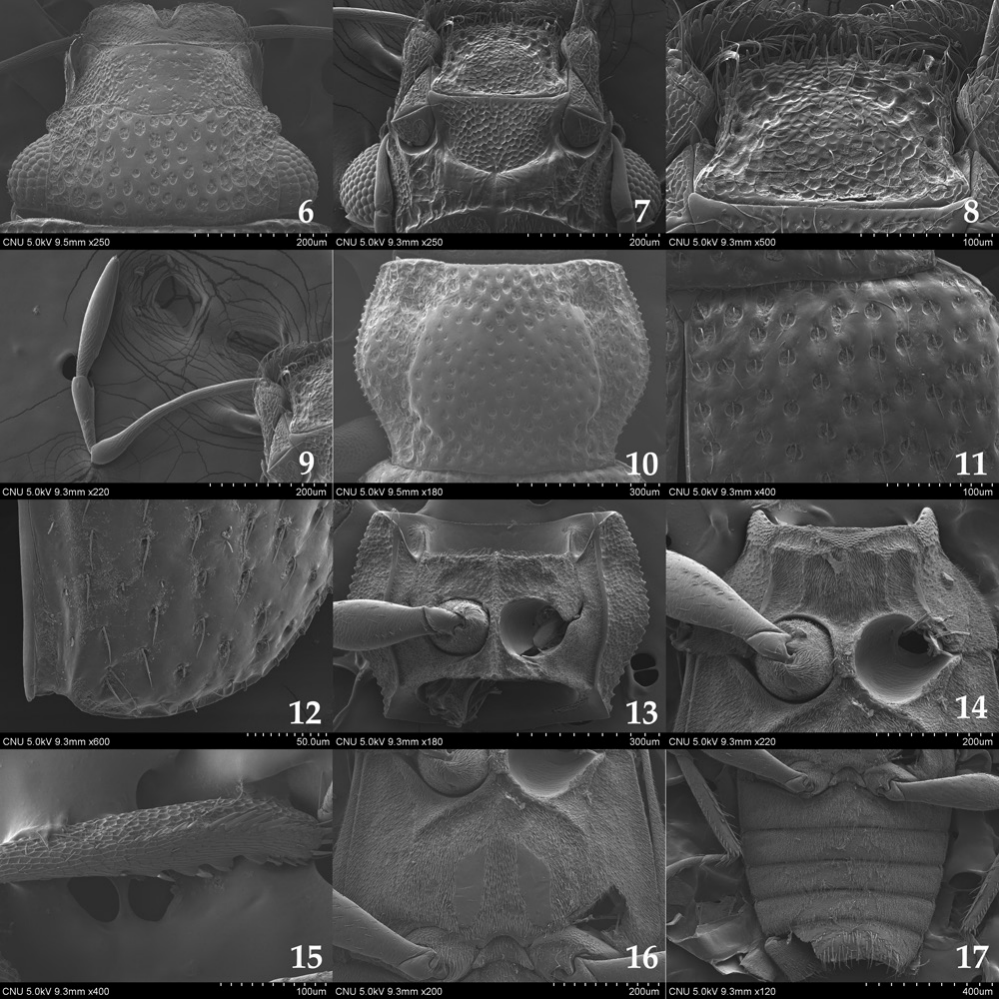
*Hydraena puetzi*. **6** head (dorsal aspect) **7** head (ventral aspect) **8** mentum (ventral aspect) **9** maxillary palpus (ventral aspect) **10** pronotum (dorsal aspect) **11** anterior part of elytron (dorsal aspect) **12** posterior part of elytron (dorsal aspect) **13** prosternum (ventral aspect) **14** mesoventrite (ventral aspect) **15** mesotibia in male (dorsal aspect) **16** metaventrite (ventral aspect) **17** abdominal sternites (ventral aspect).

##### Distribution.

Korea, China (Liaoning, Shandong), Russia (Far East).

##### Biological note.

They are found under leaves or stones in margins of mountain streams. Some specimens were collected from submerged branch and leaves in seepage on small cliff.

##### Remarks.

Kwon and Suh (1986) first recorded this species as *Hydraena riparia* in Korea. After that, many entomologists [Kim et al. (1994); Lee (1995); Han et al. (2007); Han et al. (2008); Cho and Park (2010)] reported the species, only in the local fauna without any taxonomic comments. After studying the specimen (1♂, Samcheok-si, Gagok-stream, 25.VI.1985. SH Lee) previously examined by Kwon and Suh (1986), we found that it had been incorrectly identified and actually is *Hydraena puetzi*. This species can be distinguished from *Hydraena riparia* by left paramere (Fig. 7A) parallel at middle, and apical part of right paramere (Fig. 7A) semicircular, with compact long setae on apex.

#### Subgenus Hydraenopsis

Taxon classificationAnimaliaColeopteraHydraenidae

Janssens, 1972

Hydraenopsis Janssens, 1972: 254. Type species: Hydraenopsis vietnamensis Janssens, 1972.

##### Diagnosis.

Transverse anterior inner gular carina absent; mesosternal intercoxal process protruding from mesosternal disc at an angle of 150°–180°; at least one of the two parameres shifted (Jäch et al. 2000).

#### Hydraena (Hydraenopsis) miyatakei

Taxon classificationAnimaliaColeopteraHydraenidae

Satô, 1959

Fig. 55

Hydraena miyatakei Satô, 1959: 62; Hydraena (Hydraenopsis)miyatakei: Jäch and Skale 2015: 143.

##### Specimens examined.

**SOUTH KOREA**: Gangwon Prov.: 1♀, Pyeongchang-gun, Baesujang, 29 VII 1994, SH Lee.

##### Published South Korean record.

Hydraena (Hydraenopsis) miyatakei: Jäch and Skale (2015: 143).

##### Distribution.

Korea, China (Jilin, Liaoning, Shandong), Japan, Russia (Far East).

##### Remarks.

Only one specimen of this species was colleced, not suitable for description or illustration.

#### Genus Ochthebius

Taxon classificationAnimaliaColeopteraHydraenidae

Leach, 1815

Figs 18 , 19 , 22 , 23 , 29 , 30 , 31 , 37 , 38 , 45 , 46

Ochthebius Leach, 1815: 95. Type species: Helophorus marinus Paykull, 1798.

##### Diagnosis.

Body (Fig. 26) with distinct pronoto-elytral angle. Head (Fig. 27) with a transverse groove separating clypeus and frons, the latter on each side with an interocular pit-like depression and a short longitudinal depression or groove on postero-medial part. Anterior margin of labrum truncate, sometimes with a small medial emargination. Pronotum (Figs 21, 30, 37, 45) with a narrow hyaline membranous cuticula, middle pronotal portion raised, and with longitudinal or transversal groove. Elytra (Figs 22, 31, 38, 46) with rows of punctures (Chiesa 1959; Hansen 1987; Jäch 1992).

##### Key to the species of South Korean *Ochthebius*

**Table:** 

1	Pronotum widest at anterior third to three seventh part, protruded laterally	**2**
–	Pronotum widest at middle part, rounded laterally	**5**
2	Elytra (Fig. 31) with long setae	***Ochthebius lobatus***
–	Elytra (Figs 22, 38, 46) with short setae	**3**
3	Maxillary palpomere 3 (Fig. 20) approx. 4.0 times as long as 4	***Ochthebius hasegawai***
–	Maxillary palpomere 3 (Figs 36, 44) approx. 2.0 times as long as 4	**4**
4	Medial part of metaventrite (Fig. 41) with pubescence; apical part of distal lobe of aedeagus acute	***Ochthebius marinus***
–	Medial part of metaventrite (Fig. 48) without pubescence; apical part of distal lobe of aedeagus round	***Ochthebius satoi***
5	Pronotal longitudinal groove distinct (Jäch and Delgado 2014: Fig. 6); distal lobe of aedeagus curved in lateral view (Jäch and Delgado 2014: Figs 8, 15h)	***Ochthebius ahni***
–	Pronotal longitudinal groove indistinct (Jäch and Delgado 2014: Fig. 7); distal lobe of aedeagus bisinuate in lateral view (Jäch and Delgado 2014: Figs 13, 15a)	***Ochthebius parki***

**Figures 18–25.**
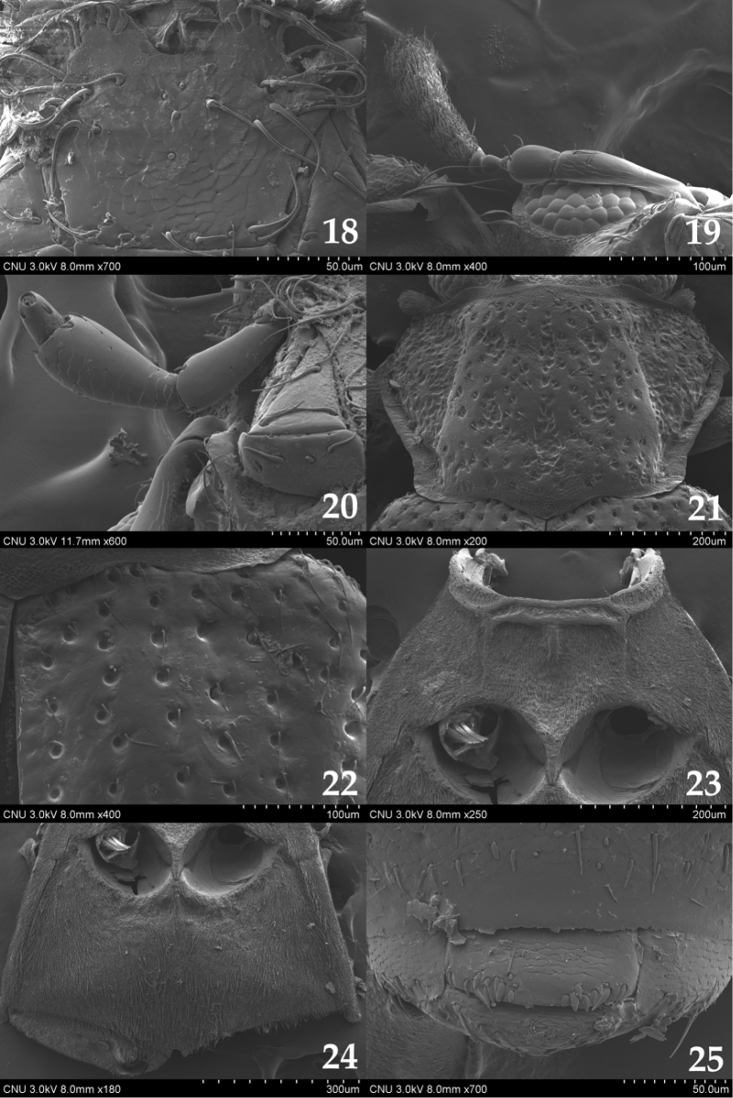
*Ochthebius hasegawai*. **18** mentum (ventral aspect) **19** antennae (lateral aspect) **20** maxillary palpus (ventral aspect) **21** pronotum (dorsal aspect) **22** anterior part of elytron (dorsal aspect) **23** mesoventrite (ventral aspect) **24** metaventrite (ventral aspect) **25** male terminal sternite (ventral aspect).

**Figures 26–33.**
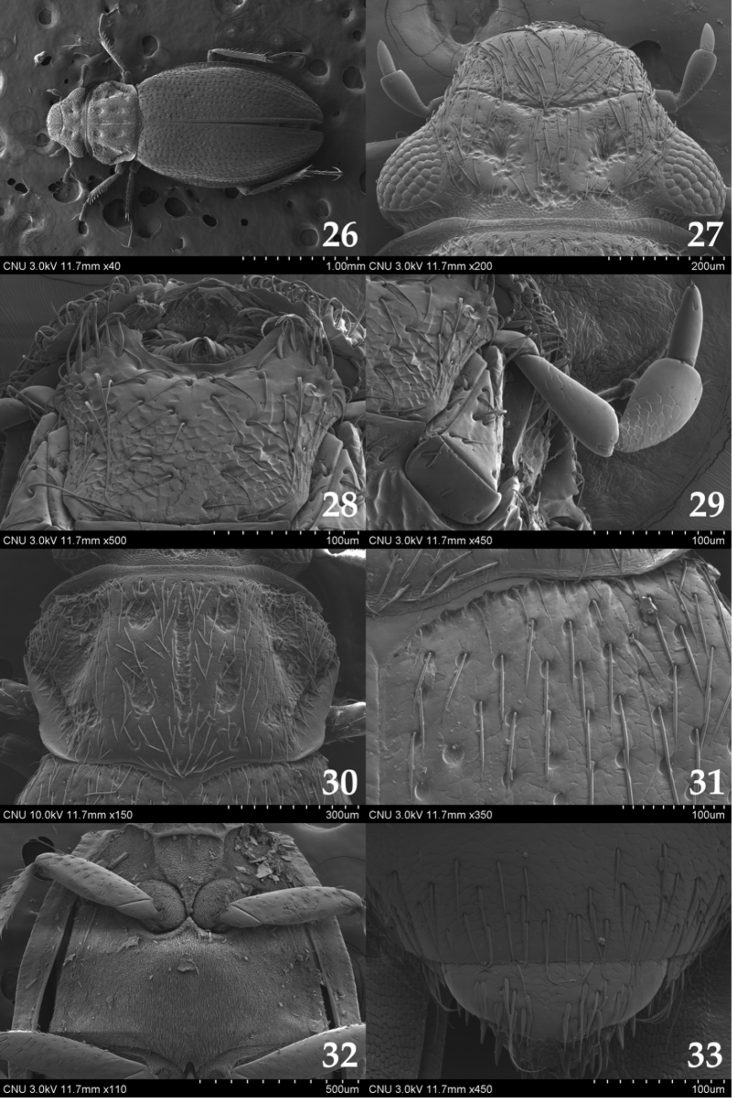
*Ochthebius lobatus*. **26** body (dorsal aspect) **27** head (dorsal aspect) **28** mentum (ventral aspect) **29** maxillary palpus (dorsal aspect) **30** pronotum (dorsal aspect) **31** anterior part of elytron (dorsal aspect) **32** meso– and metaventrite (ventral aspect) **33** sternites VIII–IX (ventral aspect).

**Figures 34–41.**
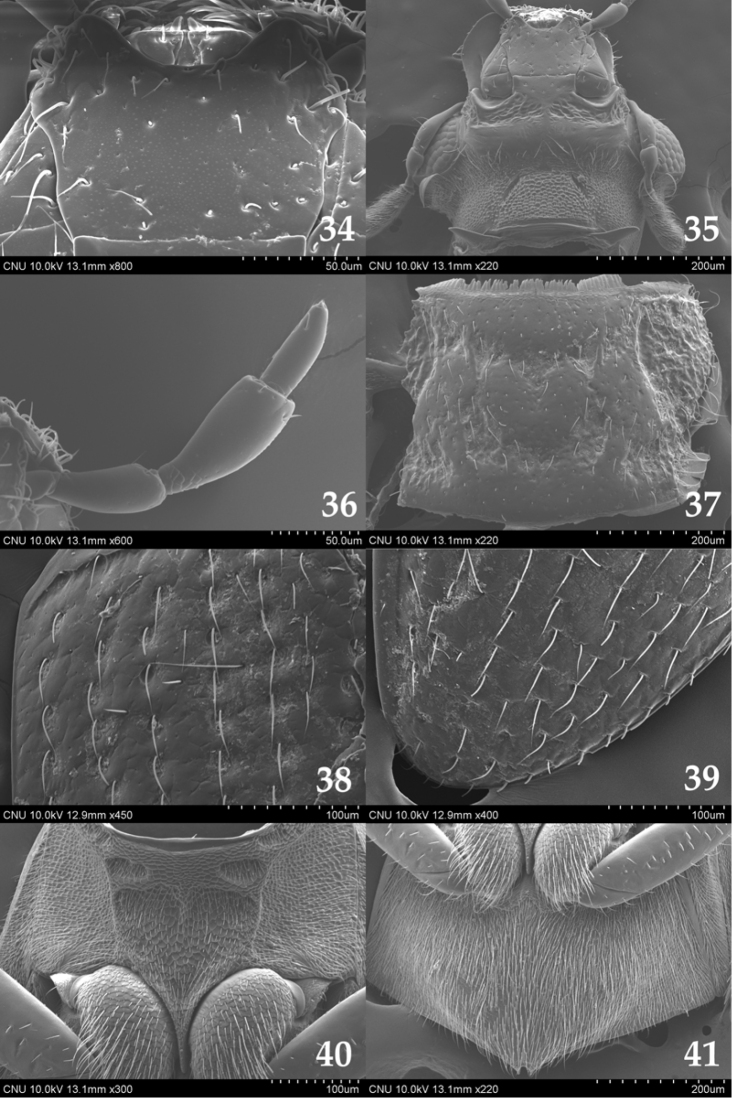
*Ochthebius marinus*. **34** mentum (ventral aspect) **35** head (ventral aspect) **36** maxillary palpus (ventral aspect) **37** pronotum (dorsal aspect) **38** anterior part of elytron (dorsal aspect) **39** posterior part of elytron (dorsal aspect) **40** mesoventrite (ventral aspect) **41** metaventrite (ventral aspect).

**Figures 42–49.**
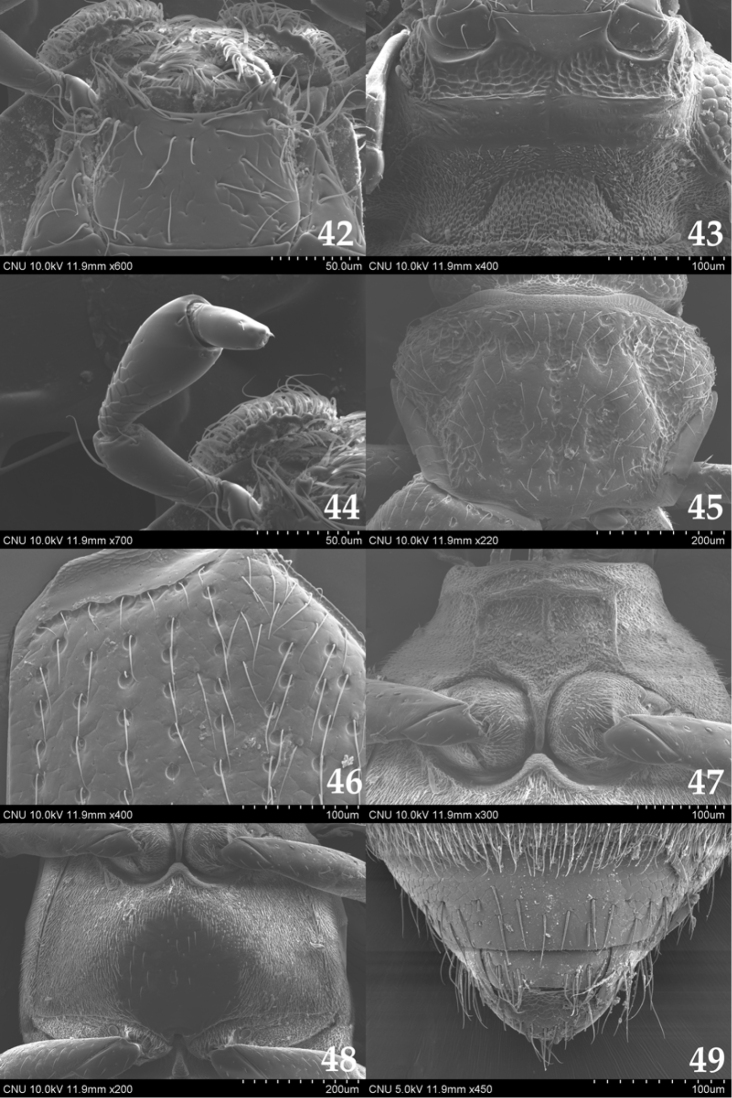
*Ochthebius satoi*. **42** mentum (ventral aspect) **43** gena and gula (ventral aspect) **44** maxillary palpus (dorsal aspect) **45** pronotum (dorsal aspect) **46** anterior part of elytron (dorsal aspect) **47** mesoventrite (ventral aspect) **48** metaventrite (ventral aspect) **49** male terminal sternite (ventral aspect).

#### Subgenus Ochthebius

Taxon classificationAnimaliaColeopteraHydraenidae

Leach, 1815

Figs 22 , 27 , 29 , 31 , 37

Ochthebius Leach, 1815: 95. Type species: Helophorus marinus Paykull, 1798. See Jäch and Skale (2015) for more detailed synonymy and references.

##### Diagnosis.

Lateral margin of pronotum (Figs 21, 30, 37, 45) with hyaline membranous cuticula. Marginal elytral ridge (Fig. 39) disappearing posteriorly. Pedicel (Fig. 19, 35) not enlarged distally (Hansen 1987; Jäch 1992).

#### Ochthebius (Ochthebius) ahni

Taxon classificationAnimaliaColeopteraHydraenidae

Jäch & Delgado, 2014

Ochthebius (*s. str.*) ahni Jäch & Delgado, 2014: 85; Jäch and Skale 2015: 153.

##### Specimens examined.

Holotype: 1♂ (CNUIC), with labels as follows: “KOREA: Gyeongbuk prov.: Kugae, 6 VII 1991, K. J. Ahn, *ex.*, rock crevice., Holotype *Ochthebius ahni* sp. n. Jäch and Delgado 2014" . Paratypes: 1♂1♀, same data as holotype. 3♂♂ 2♀♀, same data as holotype.

##### Published South Korean records.

*Ochthebius ahni*: Jäch and Delgado (2014; 85); Jäch and Skale (2015: 153). *Neochthebius granulosus*: Park and Ahn (2008: 2506) [misidentification].

##### Diagnosis.

See Jäch and Delgado (2014).

##### Distribution.

Korea.

#### Ochthebius (Ochthebius) hasegawai

Taxon classificationAnimaliaColeopteraHydraenidae

Nakane & Matsui, 1986

Figs 2 , 18–25 , 51 , 56

Ochthebius (*s. str.*) hasegawai Nakane & Matsui, 1986: 81; Jäch 1998: 186; Jäch and Skale 2015: 156.
Ochthebius mamagri Shatrovsky, 1989: 263.

##### Specimens examined.

**SOUTH KOREA**: Chungnam Prov.: 1♀, Nonsan-si, Beolgok-myeon, Sajeong-ri, N36°11'41.77" E127°16'18.77" 118 m, 24 V 2014, IS Yoo, under stone in stream; Gyeongbuk Prov.: 6♂♂ 8♀♀, Uljin-gun, Giseong-myeon, Dacheon-ri, Giseong-stream 17 VII 1995, SH Lee, mountain stream (3♂2♀, on slides); Gyeonggi Prov.: 1♀, Gapyeong-gun, Buk-myeon, Dodae-ri, Seungcheon-temple, 5 VII 2013, SW Jeong, HJ Park, stream.

##### Published South Korean record.

*Ochthebius hasegawai*: Jäch and Skale (2015: 156).

##### Redescription.

Length 1.6–2.0 mm. Body dark blue to black. Mentum (Fig. 18) 1.2 times as wide as long and with sparse setae; protruded and widest at anterior third; with a row of long setae on anterior margin. Anterior gena without long setae on posterior part in ventral view. Antenna (Fig. 19) with nine antennomeres; 1 longest, approx. 6.0 times as long as 2, two long setae present on lateral part; 2 widest at base; 3 bulbous at apical part; 4 semicircular; 5–9 clubbed and with pubescence. Maxillary palpomere (Fig. 20) 1 small, approx. 2.0 times as long as wide; 2 bulbous at apical part, 2.0 times as long as 1; 3 largest, bulbous at apical part, slightly longer than 2; 4 slender and paralleled, 0.25 times as long as 3. Pronotum (Fig. 21) reverse trapezoidal, widest at anterior fourth, with indistinct longitudinal groove on medial part, two small oval grooves on anterior part, relatively large oval groove on posterior part; anterior margin bisinuate; antero-medial margin protruded; anterior corner rectangular; lateral margin protruded at anterior third; posterior corner obtuse; postero-medial part rounded. Elytra (Fig. 22) widest at middle, with setae. Mesoventrite (Fig. 23) pentagonal, with T-shaped carina on anterior part; anterior margin transverse. Metaventrite (Fig. 24) with pubescence on medial part. Sternite VIII (Fig. 25) with more or less long setae on posterior part. Male terminal sternite (Fig. 25) semicircular and with a row of compact setae on posterior part. Median lobe of aedeagus (Fig. 51) long, slender, very weakly curved at middle; apical part acute; distal lobe slender, slightly acute apically. Paramere (Fig. 51) very short, with long setae on apical part.

**Figures 50–54.**
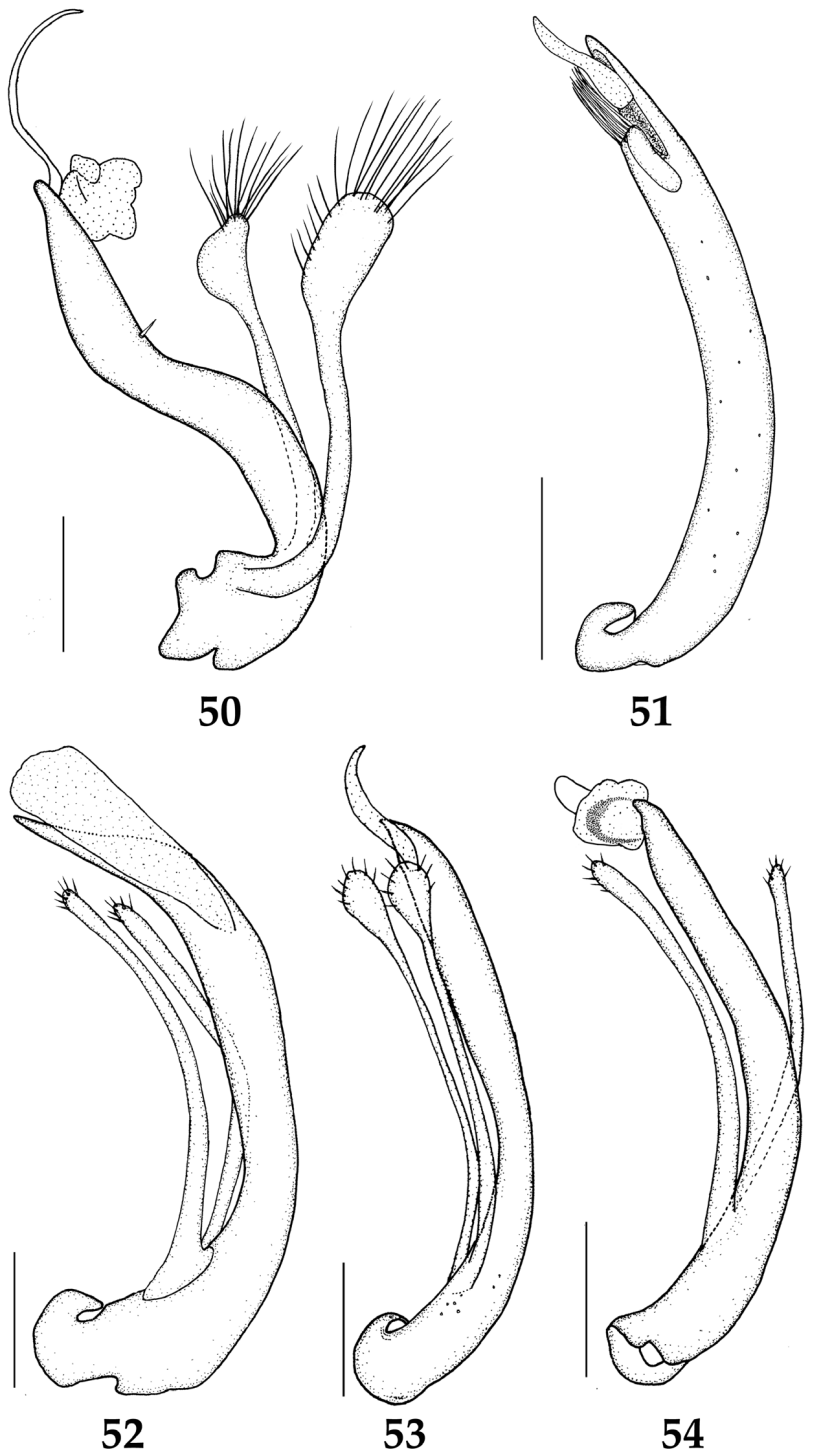
Aedeagus (lateral aspect). **50** *Hydraena puetzi* **51** *Ochthebius hasegawai* **52** *Ochthebius lobatus* **53** *Ochthebius marinus* **54** *Ochthebius satoi*. Scale bars 0.1 mm.

##### Distribution.

Korea, Japan, Russia (Far East).

**Figures 55–56.**
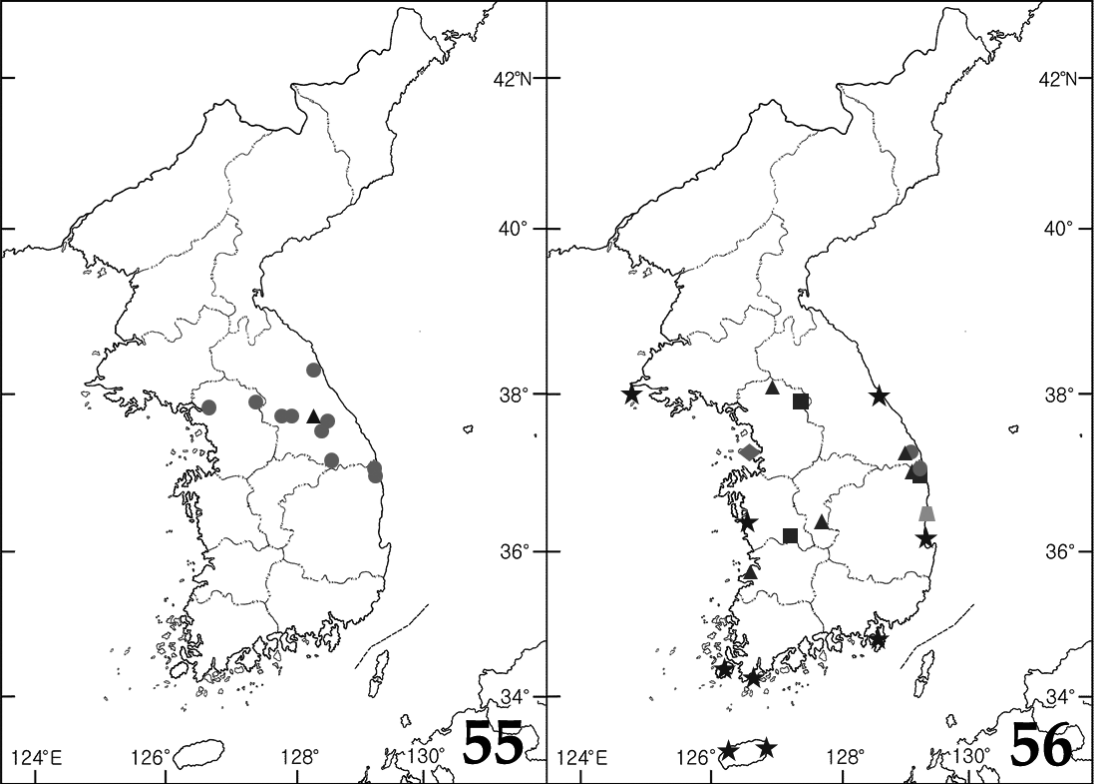
Distribution maps. **55** *Hydraena puetzi* (circle), *Hydraena miyatakei* (triangle) **56** *Ochthebius ahni* (trapezoid), *Ochthebius lobatus* (circle), *Ochthebius marinus* (diamond), *Ochthebius parki* (star), *Ochthebius satoi* (triangle), *Ochthebius hasegawai* (square).

##### Biological note.

The specimens were collected under boulders in a fast-flowing stream.

#### Ochthebius (Ochthebius) lobatus

Taxon classificationAnimaliaColeopteraHydraenidae

Pu, 1958

Figs 3 , 26–33 , 52 , 56

Ochthebius (*s. str.*) lobatus Pu, 1958: 256; Jäch 1995: 178; 2003: 351; Jäch and Skale 2015: 157.

##### Specimens examined.

**SOUTH KOREA**: Gangwon Prov.: 4♂♂ 2♀♀, Samcheok-si, Wondeok-eub, Wolcheon-ri, Gagok-steam, 1 V 1994, SH Lee, beside stream (4♂1♀, on slides); 5♂♂ 7♀♀, same data as former except for, 1 VI 1995; 1♀, same data as former except for, 25 VI 1995; Gyeongbuk Prov.: 2♂♂ 3♀♀, Uljin-gun, Namdae-stream, 17 VII 1995, SH Lee.

##### Published South Korean records.

*Ochthebius lobatus*: Jäch and Skale (2015: 157). *Ochthebius inermis*: Lee (1995: 13) [misidentification].

##### Redescription.

Length 2.2–2.6 mm. Head dark brown; pronotum and elytra brown to dark brown; ventral surface mostly brown. Dorsum (Figs 27, 30, 31) with whitish long setae. Mentum (Fig. 28) 1.2 times as wide as long and with sparse setae, widest anterior third; antero-lateral part protruded. Anterior gena without long setae on posterior part in ventral view. Antenna with nine antennomeres; 1 longest, approx. 6.0 times as long as 2, two long setae present on lateral part; 2 bulbous at medial part; 3 bulbous at apical part; 4 semicircular; 5–9 clubbed and with pubescence. Maxillary palpomere (Fig. 29) 1 small, approx. 2.0 times as long as wide; 2 bulbous at apical part, 3.5 times as long as 1; 3 bulbous at apical part, as long as 2; 4 slender and parallel-sided, 0.5 times as long as 3. Pronotum (Fig. 30) reverse trapezoidal, widest at anterior three seventh, with distinct longitudinal groove on medial part, two rounded grooves on anterior part, two oval grooves on posterior part; anterior margin bisinuate; antero-median margin straight; anterior corner rectangular; posterior corner obtuse; postero-medial part rounded. Elytra (Fig. 31) widest at middle, with long setae. Mesoventrite (Fig. 32) pentagonal with T-shaped carina on anterior part; anterior margin transverse. Metaventrite (Fig. 32) with pubescence on medial part. Sternite VIII (Fig. 33) with more or less long setae on posterior part. Male terminal sternite semicircular with few spines on posterior part. Median lobe of aedeagus (Fig. 52) weakly slender, curved; subapical part vented, inflated at anterior third; apical part acute with few setae; distal lobe cylindrical, approx. 0.25 times as long as median lobe. Paramere (Fig. 52) slender, shorter than median lobe, with few setae on apical part.

##### Distribution.

Korea, China (Chongqing, Jilin, Liaoning, Sichuan, Yunnan).

##### Remarks.

Lee (1995) first reported *Ochthebius inermis* Sharp in South Korea but we found that this was a misidentification of *Ochthebius lobatus*, based on our examination of his voucher specimens (4♂♂ 2♀♀, Samcheok-si, Wondeok-eub, Wolcheon-ri, Gagok-steam, 1 V 1994, SH Lee, ex. beside stream). This species can be distinguished from *Ochthebius inermis* by elytra brown and distal lobe of aedeagus robust.

#### Ochthebius (Ochthebius) marinus

Taxon classificationAnimaliaColeopteraHydraenidae

(Paykull, 1798)

Figs 4 , 34–41 , 53 , 56

Helophorus marinus Paykull, 1798: 245.
Ochthebius pallidus Mulsant, 1844: 61.
Ochthebius holmbergi Mannerheim, 1853: 166.
Ochthebius subabruptus Rey, 1885: 23.
Ochthebius (s. str.) marinus Jäch, 1992: 112; 2003: 330; Jäch and Skale 2015: 157.

##### Specimens examined.

**SOUTH KOREA**: Gyeonggi Prov.: 2♂♂ 3♀♀, Ansan-si, Danwon-gu, Daebudo-dong, Dongju-salt farm, N37°14'05.60" E126°36'19.55" 4 m, 7 XI 2013, DH Lee, IS Yoo, SG Lee, saline pond near salt farm (1♂, on slide).

##### Redescription.

Length 1.7–2.0 mm. Head metallic dark green; pronotum mostly pale yellowish brown and medial part brown; elytra pale yellow and rows of serial punctures dark brown; ventral surface mostly dark brown. Mentum (Fig. 34) as long as wide, widest anterior third and with sparse setae; antero-medial part broadly excised; antero-lateral part protruded. Anterior gena (Fig. 35) with long setae on posterior part in ventral view. Gula (Fig. 35) with spiny microreticulation. Antenna (Fig. 35) with nine antennomeres; 1 longest, approx. 6.0 times as long as 2, with two long setae on lateral part; 2 widest at base; 3 bulbous at apical part; 4 dish-shaped; 5–9 clubbed with pubescence. Maxillary palpomere (Fig. 36) 1 small, approx. 2.0 times as long as wide; 2 bulbous at apical part, 4.0 times as long as 1; 3 largest, bulbous at apical part, slightly longer than 2; 4 slender and paralleled, 0.5 times as long as 3. Pronotum (Fig. 37) reverse trapezoidal, widest at anterior third, with indistinct longitudinal groove on median part; anterior margin nearly straight; anterior corner rectangular; lateral margin protruded at anterior third; posterior corner obtuse; postero-medial part rounded. Elytra (Figs 38, 39) widest at middle, with setae. Mesoventrite (Fig. 40) pentagonal, with transverse thick carina on anterior part; antero-lateral part with two grooves. Metaventrite (Fig. 41) with pubescence on medial part. Sternite VIII with more or less long setae on posterior part. Male terminal sternite semicircular and with long setae on posterior margin. Median lobe of aedeagus (Fig. 53) slightly curved; apical part acute; distal lobe approx. 0.1 times as long as median lobe. Paramere (Fig. 53) shorter than median lobe; apical part oval with setae.

##### Distribution.

**Asia**: Korea, China (Beijing, Heilongjiang), Russia (East Siberia); **Europe**: Denmark, Estonia, Finland, France, Great Britain, Germany, Ireland, Latvia, The Netherlands, Norway, Poland, Russia, Spain, Sweden; **North America**.

##### Biological note.

The specimens were collected in saline pond (salinity 32.84‰) with algae and plentiful vegetation near salt farm. They were found with *Hydroglyphus coreanus* Lee & Ahn (Dytiscidae), *Berosus lewisius* Sharp, *Berosus spinosus* (Steven), *Enochrus simulans* (Sharp), and *Paracymus aeneus* (Germar) (Hydrophilidae).

##### Remarks.

This species is recorded for first time in Korea.

#### Ochthebius (Ochthebius) parki

Taxon classificationAnimaliaColeopteraHydraenidae

Jäch & Delgado, 2014

Ochthebius (*s. str.*) parki Jäch & Delgado, 2014: 88; Jäch and Skale 2015: 158.

##### Specimens examined.

Holotype: 1♂ (CNUIC), with labels as follows: “Geoje City, Gabae-ri, 1 VII 2000, K.-J. Ahn, H.-J. Kim, M.-J. Jeon, on barnacles, Holotype *Ochthebius parki* sp. n. Jäch and Delgado 2014" . Paratypes: 2♂♂, same data as holotype. **SOUTH KOREA**: Chungnam Prov.: 1♂, Boryeong-si, Ungcheon-eup, Doksan-ri, Holmoi beach, 6 IX 2003, K J Ahn, J S Park, under seaweeds; Gangwon Prov.: 1♀, Sokcho-si, Dongmyeong-dong, N38°12'48.0" E128°36'05.9" -1 m, 28 V 2012, JH Song, under stone in beach; Gyeongbuk Prov.: 3♂♂ 4♀♀, Yongyeon [=Pohang-si, Buk-gu, Heunghae-eub, Yonggok-ri], 20 VII 1991, K. J. Ahn, , in rock crevice; Gyeonggi Prov.: 1♂1♀, Incheon-City, Is. Baekryeongdo, 7 VIII 2000, C.-W. Shin, on rock (near sea); Gyeongnam Prov.: 5♂♂ 2♀♀, Geoje City, Gabae-ri, 1 VII 2000, K.-J. Ahn, H.-J. Kim, M.-J. Jeon, *ex* barnacles; 2♂♂ 3♀♀, Is. Geojedo, Dongbu-myeon, Gabae-ri, 30 VI 2001, S.-J. Park, inside barnacles; 1♂1♀, Koje, Kabae, Korean Marine Biological Laboratory, 3 VII 1998, J-Y Lyu, H-J Kim, empty barnacle; 1♂1♀, Kŏje-City, Gabae-ri, 30 VII 1999, K.-J. Ahn, on rock; Jeju Prov.: 1♂, Gwagji beach [=Jeju-si, Aewol-eub, Gwakji-ri, Gwakji beach], 3 VII 1991, K. J. Ahn, , on rock with clam; 2♂♂ 1♀, Namjeju-gun, Seongsan-eub, Ilchulbong, 11 VI 2005, S.I. Lee, S.J. Park, K.J. Ahn, M.J. Jeon, D.H. Lee, *ex* stones; Jeonnam Prov.: 2♂♂ 5♀♀, Jindo, Imhoe-myeon, Geumgab beach, 21 VIII 2001, S.-J. Park, on barnacles; 3♂♂, Wando, Sinji-myeon, Myeongsasimri beach, 23 VIII 2001, K.-J. Ahn, J.-H. Ahn, on barnacles.

##### Published South Korean records.

*Ochthebius parki*: Jäch and Delgado (2014: 88); Jäch and Skale (2015:158). *Neochthebius granulosus*: Park and Ahn (2008: 2506) [misidentification].

##### Diagnosis.

See Jäch and Delgado (2014).

##### Distribution.

Korea.

#### Ochthebius (Ochthebius) satoi

Taxon classificationAnimaliaColeopteraHydraenidae

Nakane, 1965

Figs 5 , 42–49 , 54 , 56

Ochthebius (*s. str.*) satoi Nakane, 1965: 51; Jäch 1991: 77; 1998: 177; 2003: 328; Jäch and Skale 2015: 159.

##### Specimens examined.

**SOUTH KOREA**: Chungbuk Prov.: 2♂♂ 4♀♀, Okcheon-gun, Okcheon-eub, Gyodong-ri, 4 V 1990, SH Lee, beside stream; Gangwon Prov.: 3♂♂ 5♀♀, Samcheok-si, Wondeok-eub, Wolcheon-ri, Gagok-steam, 1 VI 1995, SH Lee, beside stream (3♂♂ 1♀, on slides); Gyeongbuk Prov.: 10♂♂ 7♀♀, Uljin-gun, Giseong-myeon, Dacheon-ri, Giseong-stream 17 VII 1995, SH Lee, ex. mountain stream (2♂♂ 2♀♀, on slides); 2♂♂ 1♀, Uljin-gun, Wonnam-myeon, Gilgok-ri, Maehwa-stream, beside stream (2♂♂, on slides); Gyeonggi Prov.: 1♀, Yeoncheon-gun, Jangnam-myeon, Wondal-ri, Samicheon-bridge, 5 VII 2014, SW Jeong, stream; Jeonbuk Prov.: 1♀, Buan-gun, Byeonsan-myeon, Junggye-ri, Jikso-fall, 11 VII 2014, SW Jeong, mountain stream.

##### Published South Korean records.

*Ochthebius satoi*: Lee (1995: 14); Jäch and Skale (2015: 159).

##### Redescription.

Length 1.6–1.8 mm. Head dark brown; pronotum and elytra yellowish brown; ventral surface mostly brown. Mentum (Fig. 42) slightly longer than wide and with sparse setae; antero-lateral part protruded. Anterior gena (Fig. 43) without long setae on posterior part in ventral view. Antenna with nine antennomeres; 1 longest, approx. 6.0 times as long as 2, two long setae present on lateral part; 2 bulbous at middle; 3 oval, widest at middle; 4 dish-shaped; 5–9 clubbed and with pubescence. Maxillary palpomere (Fig. 44) 1 small, approx. 2.0 times as long as wide; 2 bulbous at apical part, 3.0 times as long as 1; 3 largest, bulbous at apical part, slightly longer than 2; 4 slender and paralleled, 0.5 times as long as 3. Pronotum (Fig. 45) reverse trapezoidal, widest at anterior third, with indistinct longitudinal groove on medial part, two small oval grooves on anterior part, relatively large oval groove on posterior part; anterior margin bisinuate; antero-medial margin straight; anterior corner rectangular; lateral side protruded at anterior third; posterior corner obtuse; postero-medial part rounded. Elytra (Fig. 46) widest at middle, with setae. Mesoventrite (Fig. 47) pentagonal, with T-shaped carina on anterior part; anterior margin transverse. Metaventrite (Fig. 48) without pubescence on medial part. Metatrochanter with a row of setae. Sternite VIII (Fig. 49) with more or less long setae on posterior part. Male terminal sternite (Fig. 49) semicircular, with sparse setae, relatively long setae on posterior part. Median lobe of aedeagus (Fig. 54) slender, curved; apical part strongly vented; distal lobe rounded. Paramere (Fig. 54) nearly parallel-sided, apical margin rounded, with few setae.

##### Distribution.

Korea, Japan, China (Henan, Jilin, Liaoning, Nei Mongol, Shaanxi, Shandong, Taiwan), Russia (Far East).

## Discussion

This study revises the number and taxonomic status of hydraenid species known to occur in South Korea. In total, eight species are recognized. Two species (*Ochthebius ahni* and *Ochthebius parki*) are apparently endemic to the South Korean fauna (Jäch and Delgado 2014). Of the remaining species, five are widespread in the East Palaearctic region (*Hydraena puetzi*, *Hydraena miyatakei*, *Ochthebius lobatus*, *Ochthebius hasegawai*, *Ochthebius satoi*) and one occurs in the Holarctic region (*Ochthebius marinus*).

Diversity of South Korean Hydraenidae is very low compared to other adjacent countries, such as China (82 endemic species out of 100 species), Japan (29 endemic species out of 35 species), and the Far East Russia (no endemic species out of 11 species) (Jäch 2003; Jäch and Díaz 2003; 2004; 2005; 2006; 2012; Jäch and Delgado 2014; Jäch and Skale 2015). They show high diversity and endemism because of their small size and limited dispersal abilities (Jäch and Balke 2008). Further collecting efforts in the Korean Peninsula will probably add more hydraenid species to the Korean fauna such as the Palaearctic species, *Hydraena riparia* Kugelann and *Ochthebius angusi* Jäch, and more species.

## Supplementary Material

XML Treatment for Hydraena

XML Treatment for Hydraena

XML Treatment for Hydraena (Hydraena) puetzi

XML Treatment for Hydraenopsis

XML Treatment for Hydraena (Hydraenopsis) miyatakei

XML Treatment for Ochthebius

XML Treatment for Ochthebius

XML Treatment for Ochthebius (Ochthebius) ahni

XML Treatment for Ochthebius (Ochthebius) hasegawai

XML Treatment for Ochthebius (Ochthebius) lobatus

XML Treatment for Ochthebius (Ochthebius) marinus

XML Treatment for Ochthebius (Ochthebius) parki

XML Treatment for Ochthebius (Ochthebius) satoi
